# The Development of Novel Primer Sets to Specifically Amplify Each of the Five Different *Deltapapillomaviruses* That Cause Neoplasia after Cross-Species Infection

**DOI:** 10.3390/vetsci8100208

**Published:** 2021-09-26

**Authors:** John S. Munday, Kristene Gedye, Cíntia Daudt, Flavio Chaves Da Silva

**Affiliations:** 1 School of Veterinary Science, Massey University, Palmerston North 4410, New Zealand; k.gedye@massey.ac.nz; 2 Laboratório de Virologia Geral e Parasitologia, Universidade Federal do Acre, Rio Branco 69920-900, Brazil; cintiadaudt@gmail.com (C.D.); veterinarioflavio@gmail.com (F.C.D.S.)

**Keywords:** papillomavirus, equine sarcoids, feline sarcoids, PCR primers, deltapapillomavirus, bovine papillomavirus

## Abstract

Bovine papillomavirus (BPV) types 1 and 2 are recognized as the main cause of equine sarcoids. However, some studies report that up to a quarter of these tumors do not contain detectible BPV1 or BPV2 DNA. The absence of detectible BPV1 or BPV2 in these sarcoids suggests the possible involvement of other papillomavirus types. Currently, five *deltapapillomaviruses* are recognized to cause mesenchymal neoplasia after cross-species infection. In addition to BPV1 and BPV2, BPV13 has been associated with equine sarcoids in Brazil, BPV14 has been associated with feline sarcoids, and Ovis aries papillomavirus 2 caused a sarcoid-like lesion in a pig. To investigate the cause of equine sarcoids, PCR primers were developed to specifically amplify each of the five different *deltapapillomaviruses* that have been associated with mesenchymal neoplasia. The specificity of these primers was confirmed using samples of formalin-fixed tissue known to contain each PV type. These primers allow rapid and sensitive detection of *deltapapillomavirus* DNA in equine sarcoids. As studies have revealed marked regional variability in the cause of equine sarcoids, these primers will be useful to determine the predominant PV type causing sarcoids in a region. Additionally, there is a single report describing mixed infections by BPV1 and BPV2 in equine sarcoids. The specific primer sets are expected to enable more sensitive detection of mixed infections in equine sarcoids. Determining the cause of equine sarcoids is important as vaccines are developed to prevent these common malignant neoplasms.

## 1. Introduction

Papillomaviruses (PVs) are double-stranded circular DNA viruses that infect most vertebrate species [1]. PVs are able to promote cell growth and replication, and PV infection can result in the development of a hyperplastic papilloma (wart) [2]. Additionally, PVs can promote the development of irreversible changes within the cell DNA and the development of neoplasia [2]. PVs are classified using the highly conserved L1 gene. If two PVs have 65–90% similarity, then they are considered to be different types, while PVs that have around 60% similarity are usually classified within the same genus [3]. In addition to the genetic similarity, PV types within the same genus often infect closely related host species and cause similar lesions [3].

Equine sarcoids are common mesenchymal neoplasms that are caused by cross-species infection by PV types that are classified within the *deltapapillomavirus* genus. Current evidence suggests that the majority of equine sarcoids are caused by bovine papillomavirus (BPV)1 or BPV2 [4], although there appear to be significant geographic differences in the proportion of equine sarcoids caused by each BPV type [5,6]. However, multiple studies have reported that around 25% of equine sarcoids did not contain amplifiable BPV1 or BPV2 DNA [7,8]. The failure to detect BPV1 or BPV2 DNA could be due to DNA fragmentation in the sample or histological misclassification of the lesions. However, it could also suggest that *deltapapillomavirus* types other than BPV1 or BPV2 could be present in a minority of tumors. The possibility of other PV types as a cause of equine sarcoids was recently demonstrated by the detection of BPV13 in an equine sarcoid from Brazil [9]. In addition to BPV1, BPV2, and BPV13, which have previously been associated with equine sarcoids, there are two additional *deltapapillomaviruses* that have been reported to cause mesenchymal neoplasia after cross-species infection. These are BPV14, which is the cause of feline sarcoids [10,11], and Ovis aries papillomavirus (OaPV)2, which was reported to cause a sarcoid-like lesion in a pig [12]. While neither BPV14 nor OaPV2 have previously been reported to infect horses, it is possible that one or both of these *deltapapillomavirus* types could cause some of the PV-negative equine sarcoids reported in earlier studies. Furthermore, there is a single report describing mixed infections by both BPV1 and BPV2 in equine sarcoids [6]. Further studies are required to determine if mixed infections with multiple *deltapapillomavirus* types are present in equine sarcoids more frequently than currently recognized.

Current research suggests that vaccines may be able to prevent equine sarcoids [13,14]. However, evidence from studies on human PV vaccines suggests that immunity against one PV type may not provide adequate cross-protection against infection by other PV types [15]. For this reason, human PV vaccines contain multiple different virus-like particles (VLPs), with each of the VLPs providing protection against one PV type [16]. While the ability of a BPV1 VLP vaccine to provide cross-protection against infection by other *deltapapillomaviruses* is currently unclear, a vaccine containing BPV1 VLPs provided good protection against BPV1 infection, but a vaccine containing both BPV1 and Equus caballus papillomavirus type 2 VLPs did not completely prevent infection by BPV2 [13]. As a vaccine designed to prevent infection by one PV type may not provide sufficient cross-protection against other PV types, determining which of the five *deltapapillomavirus* types cause the majority of equine sarcoids in a geographical region is essential to develop a logical vaccination plan. The aim of this study was to develop specific primer sets to amplify each of the five *deltapapillomavirus* types that have been associated with mesenchymal neoplasia and to confirm that each primer set only amplifies DNA from the targeted PV type. Developing specific PCR primers to exclusively amplify each *deltapapillomavirus* type will allow sensitive detection of the causative PV type without the need for direct sequencing. These primers will also allow mixed infections to be more easily detected within samples. To the authors’ knowledge, no specific primers have been previously developed to differentiate between the five *deltapapillomavirus* types that have been associated with mesenchymal neoplasia.

## 2. Materials and Methods

### 2.1. Samples and DNA Extraction

Paraffin tissue blocks containing formalin-fixed samples of an equine sarcoid that contained BPV1 DNA, a bovine anal fibropapilloma containing BPV2 DNA [17], a feline sarcoid that contained BPV14 DNA, and a sarcoid-like lesion from the mouth of a pig known to contain OaPV2 DNA [12] were used in the study. All were diagnostic samples submitted from animals in New Zealand. No positive control for the BPV13 primer set was available. Initially, the hope was to use a BPV13-positive papilloma from Brazil; however, because of COVID-19 disruptions to international freight, this was not possible. In an attempt to find a sample that contained BPV13 DNA, formalin-fixed paraffin-blocked samples of 14 cutaneous papillomas from cows in New Zealand were identified by searching the Massey University Pathology Database.

A 10 μm shaving of each paraffin block was used for DNA extraction. Total DNA was extracted from each shaving using a NucleoSpin DNA FFPE XS kit (Macherey-Nagel GmbH, Duren, Germany) according to the manufacturer’s instructions.

### 2.2. Primer Development

All primers were designed to amplify parts of the PV L1 gene using the Primer-BLAST tool (https://www.ncbi.nlm.nih.gov/tools/primer-blast, accessed 1 May 2021). The sequences used for primer design were MG977494.1 for the primers designed to amplify BPV1, KC878306.1 for BPV2, JQ798171.1 for BPV13, KP276343.1 for BPV14, and U83595.1 for OaPV2. The parameters used during primer development were the production of 10 primer sets to target each of the PV types, with primer sets amplifying a length of DNA between 100 and 250 bp. Once the primers were designed, the primer sequences were compared to the other four *deltapapillomavirus* sequences using Geneious Prime 2020.2.5 (https://www.geneious.com, accessed 1 May 2021). For each PV type, the primer set with the least sequence similarity to the other *deltapapillomavirus* types was selected. Additionally, the BPV1 and BPV2 primer sets were chosen to maximize the differences in length of the amplified DNA so that these could be used in a multiplex assay.

### 2.3. Amplification and Sequencing

The PCR reactions for all primer sets contained 1 x Hot FirePol Blend Master Mix with 10 mM MgCl_2_ (Solis BioDyne, Tartu, Estonia) and 0.25 μM of each primer. Each reaction volume was 20 μL, which included 2 μL template DNA. Amplification conditions were 95 °C for 15 min followed by 45 cycles of 95 °C for 1 min, 60 °C for 30 s, and 72 °C for 1.5 min. The final extension was at 72 °C for 5 min. Electrophoresis in a 1% agarose gel was used to detect the amplified DNA. Each primer set was used in five amplification reactions that included template DNA extracted from lesions known to contain BPV1, BPV2, BPV14, or OaPV2 and a no-template negative control. Amplicons were sequenced as previously described [18], and sequences were compared to those contained within GenBank (see http://www.ncbi.nlm.nih.gov/GenBank, accessed 1 May 2021) using the basic local alignment search tool (http://www.ncbi.nlm.nih.gov/blast, accessed 1 May 2021) to confirm that the primer sets amplified DNA from the expected PV type. To confirm that the primer set designed to specifically amplify BPV13 did not amplify DNA from the other PV types, amplification reactions containing the BPV13 primers and one of BPV1, BPV2, BPV14, or OaPV2 were performed. The primer set specific for BPV13 was also used to try to amplify PV DNA from the 14 bovine papillomas.

## 3. Results

Using the Primer-BLAST tool allowed the development of 5 primer sets to specifically amplify DNA from each of the five *deltapapillomavirus* types that have been associated with sarcoids and sarcoid-like lesions (Table 1).

To ensure that the primers only amplified the targeted PV type and did not have a high affinity to the other *deltapapillomavirus* types, the primer sequences were compared to the published sequences of the other PV types (Table 2).

**Figure 1:**
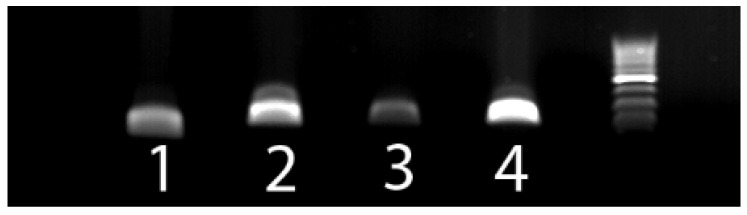
Photograph of a 1% agarose gel. Lane one is bovine papillomavirus (BPV) type 1 DNA amplified by the jmBPV1 primer set (111 bp). Lane two is BPV2 DNA amplified by the jmBPV2 primer set (198 bp). Lane 3 is BPV14 DNA amplified by the jmBPV14 primer set (195 bp). Lane 4 is Ovis aries papillomavirus type 2 DNA amplified by the jmOaPV2 primer set (219 bp). The DNA ladder contains bands at 100 bp increments, with the bright band an amplicon of 500 bp.

The primer sets designed to amplify BPV1, BPV2, BPV14, and OaPV2 all amplified PV DNA from the expected samples (Figure 1). Sequencing confirmed that the amplified DNA was from the expected PV type. None of the primer sets amplified DNA from any of the other four *deltapapillomavirus* types or from the no-template negative controls. No PV DNA was amplified by the BPV13-specific primer set from the samples known to contain BPV1, BPV2, BPV14, or OaPV2 DNA. This confirmed the specificity of this primer set. However, no positive control for these primers was available, and BPV13 DNA was not amplified from any of the bovine papillomas included in the study.

## 4. Discussion

Current evidence suggests that most equine sarcoids are caused by BPV1 or BPV2. However, there appears to be significant geographical variability in the proportions of sarcoids caused by each BPV type, with almost all equine sarcoids from Europe caused by BPV1 but the majority of North American equine sarcoids caused by BPV2 [5,6,8,19]. Currently, there is no evidence that BPV13 causes equine sarcoids in any country other than Brazil [9]. However, the detection of this PV type in bovine tissues from Brazil, Italy, and China suggests that BPV13 may have a more global distribution than previously recognized [20,21,22]. There is currently no evidence that BPV14 or OaPV2 causes equine sarcoids. However, a proportion of sarcoids do not contain detectible BPV1 or BPV2 [7,8] suggesting the possibility that a previously unrecognized PV could have caused these neoplasms. As BPV13, BPV14, and OaPV2 have been shown to cause mesenchymal neoplasia after cross-species infection [9,10,12], these PV types are potentially global causes of equine sarcoids. The use of the currently reported primer sets will allow the detection of all five *deltapapillomavirus* types that are currently known to cause mesenchymal neoplasia. This will allow rapid and simple determination of whether BPV1, BPV2, or BPV13 is the prominent cause of equine sarcoids in a region. Additionally, the primers allow sensitive detection of BPV14 and OaPV2, allowing it to be confirmed whether or not these PV types are able to infect horses and cause equine sarcoid development. Furthermore, there is a single report of both BPV1 and BPV2 DNA within a series of equine sarcoids [6]. The use of specific primers to detect each PV type allows sensitive detection of multiple different PV types within a sarcoid. Determining the causative PV types is important, as vaccines are developed to prevent these common equine neoplasms [14].

Currently, the *deltapapillomavirus* type present within an equine sarcoid is determined using one of two methods. The first method uses PCR primers that were designed to amplify both BPV1 and BPV2 DNA. Following amplification, the BPV type is then determined using restriction enzyme assays [5,6,7] or direct sequencing [8,23]. The disadvantage of using this method is that it is uncertain whether or not any PV types other than BPV1 or BPV2 would be detected. Furthermore, if the primers do amplify other PV types, these non-BPV1/ BPV2 types would only be detected using the restriction enzyme method if the enzymes cleave the PV DNA at different sites from either BPV1 or BPV2. This suggests that it is possible that BPV13, BPV14, or OaPV2 could be present within equine sarcoids but have remained undetected in previous studies. An additional disadvantage of using this technique is that it is possible that co-infections by BPV1 and BPV2 could be undetected if one BPV type is present in higher quantities within the sarcoid. This is because the BPV with the higher concentration of DNA would then be amplified to a much higher concentration, potentially masking the presence of other PV types that could be present. Whether mixed infections by PVs in equine sarcoids are truly rare or simply uncommonly recognized is currently uncertain.

The second method used to determine the type of PV present within an equine sarcoid uses consensus PCR primers to amplify DNA from a wide range of PV types followed by sequencing of the amplified DNA to determine the causative type [19,24]. As the commonly used consensus primers, such as FAP59/64 or MY09/11, are known to amplify all 5 *deltapapillomavirus* types, this method has the advantage of being able to detect a wide range of PVs that could be present within equine sarcoids [9,10,12,19]. However, both the FAP59/64 and MY09/11 primer sets amplify long (around 450 bp) DNA sequences [25,26]. Amplification of such a long sequence is generally possible when unfixed tissue is available. However, as formalin fixation causes breaks in the DNA [27], it can be hard to amplify such a long sequence from formalin-fixed tissue. As sarcoids are typically diagnosed using histology of formalin-fixed tissues, this may result in a number of sarcoids that do not contain sufficient intact DNA to be amplified by these primers. Additionally, for the causative type to be determined, it is necessary to purify and sequence each amplification product. As such sequencing is time-consuming and expensive, this makes the use of consensus primers less suitable for evaluating large numbers of lesions. Using consensus primers has the additional disadvantage of only being able to identify one PV type from each lesion. Furthermore, a PV type that has more affinity for the consensus primers is more likely to be amplified than a PV type with less affinity. This suggests that it is possible that small quantities of an incidental PV infection could be detected, while the causal PV infection could be missed if the primers have a lower affinity for this type.

The use of multiple primer sets to specifically amplify each of the potentially causative *deltapapillomavirus* types has a number of advantages. Firstly, as the specificity of each of the primer sets was demonstrated in this study, the amplification of DNA by a primer set confirms the *deltapapillomavirus* type. This saves time and expense by removing the need for subsequent direct sequencing or restriction enzyme analysis. Another advantage of using the specific primers is that mixed infections will be detected more easily, even if there are differences in the concentrations of DNA from each PV type present in the sample. The ability to detect mixed infections will be greatest if each primer set is used separately rather than in a multiplex assay. Additional studies of equine sarcoids using methods that are known to allow amplification of multiple PV types will give confidence regarding how common mixed infections are within these neoplasms. Compared to the commonly used consensus primers, only a short length of PV DNA is amplified by the specific primers. This is important, as this increases the chances that DNA will be able to be amplified, even when formalin-induced fragmentation is present. As the primers are specifically designed to amplify one PV type, the primer sequence matches exactly the targeted *deltapapillomavirus* DNA sequence. Compared to consensus primers, this allows higher annealing temperatures and higher sensitivity.

There are some disadvantages to using specific primers. Firstly, these primers will only allow identification of the five targeted *deltapapillomavirus* types, and any other PV types present within a lesion will not be detected. In addition, when using each primer set separately, five PCR reactions per sample are required. While this is not too problematic for small numbers of cases, significant numbers of reactions need to be performed to evaluate large series of sarcoids. Most sarcoids are thought to be caused by BPV1 or BPV2 [5,6,8,23]. To reduce the number of reactions required, the primers specific for BPV1 and BPV2 could be run in a multiplex assay. Additional sequencing is not required, as the amplicons are of different sizes.

If initial studies of equine sarcoids confirm most are caused by BPV1 or BPV2 and few, or none, are caused by BPV13, BPV14, or OaPV2, it would seem to be unnecessary to include all five primers sets in the routine evaluation of equine sarcoids. It is possible that the primer sets specific for BPV13, BPV14, and OaPV2 will be most valuable for evaluating equine sarcoids that do not contain either BPV1 or BPV2 DNA.

As no sample containing BPV13 could be used for the study, it cannot be confirmed that the BPV13-specific primer set amplifies BPV13 DNA. Additional testing of this primer set will be performed when a positive control is available, and an addendum will be added if these primers fail to amplify BPV13 DNA as expected. However, as the primers to amplify BPV13 were developed using identical methods to the primers that amplified BPV1, BPV2, BPV14, and OaPV2 DNA, it seems most likely that the BPV13-specific primer set would amplify BPV13 DNA as expected. Potentially more importantly, the study confirmed that the BPV13-specific primer set did not amplify BPV1, BPV2, BPV14, or OaPV2 DNA sequences. None of the bovine papillomas contained amplifiable BPV13 DNA, suggesting that infection by this PV type is rare in New Zealand cattle.

## 5. Conclusions

The methods described in this manuscript will be extremely useful for studies of the causes of equine sarcoids throughout the world. They allow rapid and simple determination of the predominant PV type to cause equine sarcoids in a geographical region. As previous studies have shown significant geographical variation in the causes of these common tumors, this information will allow rational vaccination plans to prevent these common malignant tumors of horses.

## Figures and Tables

**Table 1:** The primer sequences used to amplify the five sarcoid-associated *deltapapillomavirus* types in this study. BPV is bovine papillomavirus, OaPV is Ovis aries papillomavirus.

Target Papillomavirus	Primer	Length of Amplicon (bp)
BPV1	jmBPV1F. 5′-AGCTGTGATTTCCACAGAGC-3′ jmBPV1R. 5′-TGGAACCCCACTAACAGAGT-3′	111
BPV2	jmBPV2F. 5′-CTGTGCCTCCTAGTGGTTGG-3′ jmBPV2R. 5′-TACCAAGTCACTGTGGGGGA-3′	198
BPV13	jmBPV13F. 5′- ACAGTTGAACATCCTGCCCC-3′ jmBPV13R. 5′- ATCCCAAAACCGTAGCCCTG-3′	112
BPV14	jmBPV14F. 5′-GGAACAAACCTCACAATCAC-3′ jmBPV14R. 5′-CCAGTTCTCTAATACTGAGG-3′	195
OaPV2	jmOaPV2F. 5′-CTCGTAACCATTGCCTCATGC-3′ jmOaPV2R. 5′-TGCCAGCAACAATCAGGCTA-3′	219

**Table 2:** Summary of the similarities between each of the primer sequences and the L1 ORF sequences of each of the five *deltapapillomavirus* types that have been associated with mesenchymal neoplasia. The numerator indicates the number of identical base pairs between the primer sequence and each papillomavirus DNA, while the denominator indicates the number of bases within the primer sequence. BPV is bovine papillomavirus, OaPV2 is Ovis aries papillomavirus.

Primer	BPV1	BPV2	BPV13	BPV14	OaPV2
jmBPV1F	*20*/*20*	15/20	17/20	12/20	14/20
jmBPV1R	*20*/*20*	15/20	14/20	13/20	13/20
jmBPV2F	14/20	*20*/*20*	16/20	16/20	16/20
jmBPV2R	14/20	*20*/*20*	18/20	13/20	15/20
jmBPV13F	17/20	16/20	*20*/*20*	15/20	16/20
jmBPV13R	14/20	14/20	*20*/*20*	14/20	15/20
jmBPV14F	13/20	14/20	13/20	*20*/*20*	17/20
jmBPV14R	14/20	15/20	15/20	*20*/*20*	14/20
jmOaPV2F	15/21	13/21	14/21	13/21	*21*/*21*
jmOaPV2R	15/20	15/20	15/20	14/20	*20*/*20*

## Data Availability

The data presented in this study are available on request from the corresponding author.
